# The spread of incompatibility-inducing parasites in sub-divided host populations

**DOI:** 10.1186/1471-2148-8-134

**Published:** 2008-05-06

**Authors:** Max Reuter, Laurent Lehmann, Frédéric Guillaume

**Affiliations:** 1 Research Department for Genetics, Evolution and Environment, Faculty of Life Sciences, University College London, Wolfson House, 4 Stephenson Way, London, NW1 2HE, UK; 2 Department of Genetics, Cambridge University, Downing Street, Cambridge, CB2 3EH, UK; 3 Department of Zoology, University of British Columbia, 6270 University Blvd., Vancouver, BC V6T 1Z4, Canada; 4 Department of Biological Sciences, Stanford University, Stanford, CA, USA

## Abstract

**Background:**

Maternally transmitted symbionts have evolved a variety of ways to promote their spread through host populations. One strategy is to hamper the reproduction of uninfected females by a mechanism called cytoplasmic incompatibility (CI). CI occurs in crosses between infected males and uninfected females and leads to partial to near-complete infertility. CI-infections are under positive frequency-dependent selection and require genetic drift to overcome the range of low frequencies where they are counter-selected. Given the importance of drift, population sub-division would be expected to facilitate the spread of CI. Nevertheless, a previous model concluded that variance in infection between competing groups of breeding individuals impedes the spread of CI.

**Results:**

In this paper we derive a model on the spread of CI-infections in populations composed of demes linked by restricted migration. Our model shows that population sub-division facilitates the invasion of CI. While host philopatry (low migration) favours the spread of infection, deme size has a non-monotonous effect, with CI-invasion being most likely at intermediate deme size. Individual-based simulations confirm these predictions and show that high levels of local drift speed up invasion but prevent high levels of prevalence across the entire population. Additional simulations with sex-specific migration rates further show that low migration rates of both sexes are required to facilitate the spread of CI.

**Conclusion:**

Our analyses show that population structure facilitates the invasion of CI-infections. Since some level of sub-division is likely to occur in most natural populations, our results help to explain the high incidence of CI-infections across species of arthropods. Furthermore, our work has important implications for the use of CI-systems in order to genetically modify natural populations of disease vectors.

## Background

Vertically transmitted symbionts are common in nature. They are of diverse phylogenetic origin, comprising viruses, bacteria, protozoa, and fungi, and reside in an equally diverse range of hosts, including both animals and plants[1-5]. Many vertically transmitted symbionts live inside the host cytoplasm and accordingly their transmission is strictly maternal. For these symbionts to spread through a host population, the number of infected daughters produced by an infected female must exceed the number of daughters produced on average in the population. Symbionts have evolved a number of strategies to achieve this goal. Some, such as secondary symbionts of aphids, are beneficial and increase the host's overall fitness through a positive effect on fecundity or survival [6,7]. Others spread by biasing the sex ratio of their host towards females. This strategy is common in symbionts of arthropods, which achieve sex ratio distortion by inducing parthenogenesis, feminizing genetic males, or selectively eliminating male hosts [2,8]. A third strategy differs fundamentally from the previous two in that it promotes infection indirectly by impeding the reproduction of uninfected female hosts rather than affecting that of infected ones [8-10]. This is achieved through a mechanism called cytoplasmic incompatibility (CI) which appears to have evolved at least twice independently in two bacterial symbionts of arthropods, *Wolbachia *[11] and *Cardinium *[12]. Incompatibility is presumably caused by a modification in the sperm of infected males that causes increased zygote mortality unless rescued by a symbiont present in the egg [2]. CI therefore specifically targets the fecundity of uninfected hosts.

Cytoplasmic incompatibility benefits infected individuals by reducing the fecundity of uninfected females in a targeted way. This reduction increases the relative fitness of infected females, i.e., their fecundity measured relative to the average fecundity in the population. The degree to which CI increases the relative fitness of infected depends on the frequency of incompatible matings, making selection on CI frequency-dependent [9,13-16]. Whether CI is selectively favored depends on the balance between the increase in relative fitness of infecteds due to CI and forces that act against its spread, namely a fecundity cost of bearing an infection and imperfect transmission of the symbiont. In the presence of a cost of infection and/or imperfect transmission, an unstable infection equilibrium exists (often referred to as the 'invasion threshold'), separating the lower frequency range in which infection is counter-selected and the higher frequency range where it is selected for.

For symbionts to spread in a host population, infection frequency has to exceed the unstable equilibrium. Many authors have therefore made the verbal argument that random genetic drift would be required for the successful spread of CI-symbionts [9,17]. Egas et al. [17] tested this conjecture in computer simulations that determined the probability of CI spread in random mating populations of different sizes (and hence subject to varying levels of genetic drift). The authors concluded that random drift was unlikely to drive infection frequency beyond the invasion threshold and allow it to go to fixation. This result is at odds with the high number of species that have been found to be infected with CI-inducing parasites [see [18] for a review]. Egas et al. therefore conclude that in addition to CI, infection spread is driven by factors such as other reproductive manipulations, effects of infection on the population sex ratio or other fitness-compensating effects [17].

The conclusion of Egas et al.'s seems pessimistic for the spread of CI infection in panmictic populations of large size. However, their data also show that CI can spread with considerable probabilities when populations are small. This raises the possibility that the spread of CI might be facilitated if a large population is subdivided into a number of discrete demes that are linked by migration. Population sub-division is known to induce increased levels of local genetic drift, which could drive infection frequency beyond the invasion threshold within demes. Despite its clear relevance, the effect of genetic drift on CI spread in sub-divided population has found little treatment in the theoretical literature. Some studies have analyzed CI-dynamics either in continuous [19] or discrete space [20], but have done so using deterministic models which ignore the stochastic effect due to drift. The only model including drift (and only implicitly) has come to the surprising prediction that population structure would impede the spread of infection. In this model, Wade and Stevens [21] assume that hosts aggregate in groups to mate and reproduce before being mixed and compete at the scale of the whole metapopulation. Frequency change of CI was then calculated as a function of the variance in infection frequency between breeding groups. Wade and Stevens showed that increasing this variance, as would happen because of drift or if infection appeared in a subset of the groups, raised the invasion threshold and reduced the per-generation change in average infection frequency across groups.

Motivated by the discrepancy between biological intuition and theoretical prediction, we present here a new analysis of CI dynamics in sub-divided host populations. We first derive an analytical model which illustrates the different components of the selection pressure acting on a CI phenotype and how they are affected by population sub-division. This model allows us to derive the conditions under which a rare infection can spread in a population and to determine the expected equilibrium infection frequency. We complement the analytical model with individual-based simulations. These relax some of the simplifying assumptions made in the model and generate predictions for more complex population structures.

## Results

### Analytical model

Similar to previous models treating the panmictic case we will ignore the dynamics of symbiont populations within individual hosts and concentrate on the presence or absence of parasite infection. This allows us to conveniently model the spread of infection as a two state model in which individuals are in one of the two states 'infected' and 'uninfected'. Transmission of infection state is purely maternal, resembling symbiont transmission in natural host populations. Vertical transmission of infection is assumed to be perfect and all offspring of infected mothers are infected. This assumption is justified because natural parasites causing CI usually show high rates of vertical transmission [9], but is relaxed in simulations presented below.

We assume that hosts live in a population composed of an infinite number of demes, each containing a finite number *N *of breeding males and *N *females (i.e., infinite island model assumptions). Hosts are diploid and semelparous (no overlapping generations) and undergo the following lifecycle: (1) Adult hosts mate randomly within demes. (2) Adult hosts reproduce. Each female produces a large number of offspring that is a function of the infection status of her and her mating partner. The fecundity of an uninfected female mated to an infected male (incompatible mating), relative to that of uninfected female mated to an uninfected male, is (1 - *B*) and the relative fecundity of an infected female is (1 - *C*), independently of the genotype of her mate. In these fecundities, *C *is the fecundity cost of infection borne by infected females and *B *is the fecundity cost of cytoplasmic incompatibility borne by uninfected females mated to infected males (note that for clarity we have chosen to deviate from the usual notation in which *C *= *s*_*f *_and *B *= *s*_*h*_). All adults die after reproduction. (3) Juvenile hosts disperse. Male and female juveniles disperse with equal probabilities and randomly, meaning that any non-natal deme is reached with same probability. The probability that a juvenile is sampled in its natal deme after dispersal is given by (1 - *m*) where *m *is the migration rate. (4) Juveniles compete for access to reproduction. Competition is sex-specific and occurs within demes. In each deme, *N *juveniles of each sex reach adulthood, the remainder die. This life cycle drastically differs from that of Wade & Stevens [19], where mixing of individuals is complete after breeding and occurs at the scale of the whole population. However, our model converges towards that of Wade & Stevens at the higher migration limit *m *→ 1.

Assuming this life-cycle and weak selection (small *C *and *B*), we show in the Appendix that the change in frequency (Δ*p*) of infection in the population can be written as

(1) $$\Delta p=p(1-p)\left[-C(1-F_{st})+B{\left(1-m\right)}^{2}\left(F_{st}-F_{3}^{R}\right)+pB\left(1-g(N,m)\right)\right]$$

where *F*_*st*_, $F_{3}^{R}$, and *g*(*N*, *m*) are terms describing the effect of population sub-division on the probability that individuals in a deme share their infection status. More precisely, *F*_*st *_is Wright's measure of population structure describing the probability that two randomly sampled individuals within a deme share their infection status by descent, $F_{3}^{R}$ is the probability that one male and two females sampled with replacement from the same deme share their infection status by descent, and *g*(*N*, *m*) is a function depending on deme size and migration rate (see eq. 21 in the Appendix).

The selective pressure on infection (the term in square brackets) comprises three components. The first, -*C*(1-*F*_*st*_), describes the effect of directional selection against infection due to the fecundity cost *C *borne by infected females. The fecundity cost is weighted by the probability (1-*F*_*st*_). This weighting expresses the fact that population sub-division reduces the strength of this component of selection. Specifically, genetic drift leads to increased coalescence within demes, thereby homogenising local demes with respect to infection status and levelling differences in fecundity between competitors. The second and third components of selection capture the change in infection frequency due to the benefit of CI. These arise whenever an infected female enjoys increased relative fitness due to the reduced productivity of an uninfected females having an incompatible mating with an infected male. Accordingly, the benefit of CI is proportional not only to the cost of incompatibility, *B*, but also to the frequency with which one infected female, one infected male and one uninfected female occur in a same deme (the first being the beneficiary, the latter two the incompatible mates). Equation 1 distinguishes two cases that can lead to this constellation, based on the genetic relationship between the infected female and male. The first of the benefit terms, *B*(1-m)^2^(*F*_*st *_- $F_{3}^{R}$), is frequency-independent (does not contain *p*) and measures the beneficial effect arising from events where the infected male and female are related, i.e. are both infected due to recent coalescence. Accordingly, the term weights the cost of incompatibility, *B*, by the term (*F*_*ST *_- *F*_3_^*R*^) which quantifies the joint probability that within a deme, a female shares her infection status by recent descent with an infected male mated to an uninfected female (who will suffer from incompatibility). Local genetic drift affects the term (*F*_*ST *_- *F*_3_^*R*^) in a non-trivial way, because on one side it increases the probability that a female shares the same infection status as a male, but on the other side it decreases the probability that this male will have mated with a female bearing the alternative status. As a consequence of these opposing effects, (*F*_*ST *_- *F*_3_^*R*^) is not necessarily a monotonic function of *N *and *m*. The weighting by (1 - *m*)^2 ^expresses the fact that the offspring of an infected female have to remain in their natal patch and compete against other sedentary offspring in order for an increase in the relative fitness of infected females to arise. The second benefit term, *pB*(1-*g*(*N*, *m*)), measures the benefit of CI that is independent of recent coalescence. Here, the infected male and female are unrelated in the sense there is no recent coalescence in their infection status. Accordingly, the probability of such an event is frequency-dependent and the term includes the average frequency *p *of infection in the population. The term is further offset by a quantity *g*(*N*, *m*) which measures the effect of population sub-division on the frequency with which such a benefit occurs. The function *g*(*N*, *m*) decreases monotonically in *N *and *m *(see Appendix), reflecting the fact that with reduced population sub-division, the benefit of CI relies more and more on interactions between unrelated individuals.

Equation 1 also allows us to recover the dynamics of CI infections considered by previous treatments. When deme size becomes very large (*N *→ ∞) or when migration is complete (*m *→ 1), equation 1 reduces to

(2) Δ*p *= *p*(1 - *p*) [-*C *+ *pB*],

which is the weak-selection equivalent of the frequency change calculated by [14].

We will now consider the situation when infection is rare (*p *→ 0). This situation is highly relevant, because it is the natural condition faced by all invading CI-infections and the dynamics of infection at this point determine the probability of invasion (or, in mathematical terms, the stability of the equilibrium *p *= 0). Taking the lower frequency limit of equation 1, we can derive the condition for the invasion of a rare CI-infection in terms of the cost-to-benefit ratio *C*/*B*,

(3) $$\frac{C}{B}<{\left(1-m\right)}^{2}\frac{\left(F_{st}-F_{3}^{R}\right)}{\left(1-F_{st}\right)}.$$

**Figure 1:**
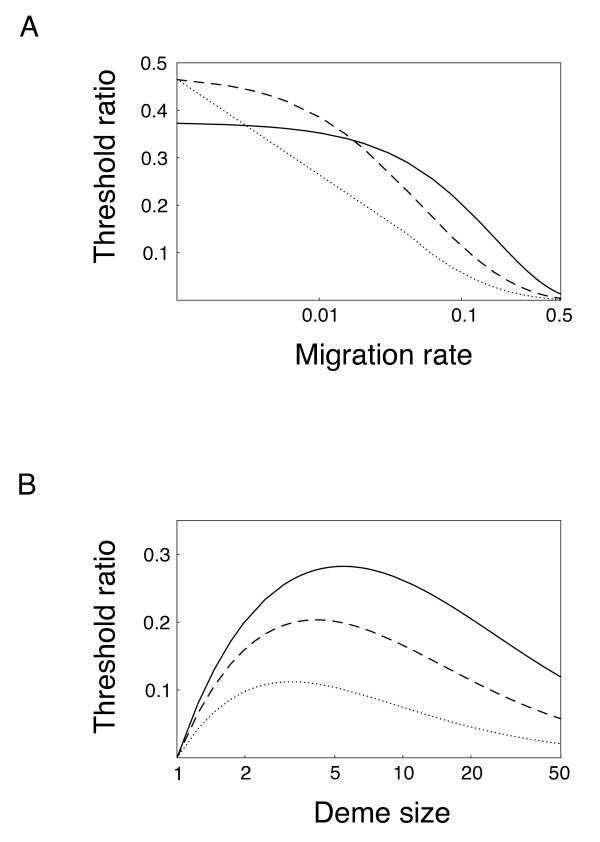
**Analytical predictions on the effect of population structure on CI-dynamics**. The figure shows the threshold cost-to-benefit ratio (C/B) for the invasion of CI-symbionts. A. Threshold ratio as a function of host migration rate for deme sizes of *N *= 4 (solid line), *N *= 20 (dashed line), and *N *= 50 (dotted line). B. Threshold ratio as a function of deme size for host migration rates of *m *= 0.05 (solid line), *m *= 0.1 (dashed line), and *m *= 0.2 (dotted line).

The expression on the right-hand size of this inequality is a function of deme size (*N*) and migration rate (*m*). Equating both sides of inequality 3, we can define a threshold cost-to-benefit ratio above which infections will not be able to invade the population. Figure 1 shows how this threshold ratio varies with both host migration rate and deme size. For population with very large deme sizes and very high migration rates (i.e., quasi-panmixia) the threshold approaches zero and infections cannot invade unless they are virtually cost-free relative to the intensity of incompatibility. With decreasing migration rate, however, the threshold ratio increases monotonically, indicating that infections can spread even if associated with a fecundity cost (C>0) and/or incomplete incompatibility (B<1). The change of the threshold ratio with deme size is non-monotonous, the threshold ratio being highest at intermediate deme sizes. This effect can be interpreted in terms of the impact of local drift on the frequency of incompatible matings. In host populations with intermediate deme size, drift maintains the local variance in infection at a level that maximizes the benefit of incompatibility, making such population most prone to the invasion of CI-infections.

### Simulations

To complement the analytical model, we ran individual-based computer simulations of CI spread, based on an extended version of the population genetics software Nemo [22]. The simulations allowed us to relax some of the more idealistic assumptions of the model and measure additional parameters of infection dynamics, such as the speed of spread and the equilibrium frequency attained.

The simulations considered a finite population of 1800 individuals (half males, half females) subdivided into of *n*_*d *_demes of *N *individuals each. The demes are linked by migration at rate *m*. We implemented two migration models, an island model (all demes equidistant) and a stepping stone model (populations arranged on a ring). Furthermore, in one set of simulations we explored the effect of differential male and female migration in an island model.

The life cycle is identical to that assumed in the analytical model. Male and female fecundity was assumed Poisson distributed and each individual was assigned a number of offspring drawn form a distribution with mean and variance *f *= 10. The cost of infection was implemented by reducing the fecundity of infected females by a factor (1 - *C*). Cytoplasmic incompatibility acted upon fertilization. An uninfected egg fertilized by the sperm of an infected father died with probability *B*. In all simulations, we relaxed the assumption of perfect vertical transmission made in the analytical model. Thus, we assumed that infection was lost with a probability μ from mother to offspring.

Throughout this study we used a standard set of infection parameters with an incompatibility cost of *B *= 0.5, a fecundity cost of *C *= 0.01, and a rate of parasite loss during vertical transmission of μ = 0.05. Based on the model for an infinite panmictic population [14], an infection with these parameters is selectively favoured when its frequency exceeds the invasion threshold of *p*_*it *_= 0.13 and will attain an equilibrium frequency of *p*_*e *_= 0.94. The parameter values used are inspired by empirical data obtained in populations of *Drosophila simulans *harbouring a CI-Wolbachia [23]. Additional simulations (data not shown) showed that varying the values has the effect expected under the deterministic model of infection dynamics with panmixia.

At the start of each simulation, one deme was inoculated with one infected female and one infected male. This starting frequency is well below the invasion threshold of *p*_*it *_= 0.13 (and hence counter-selected) when considering the whole population of 900 males and 900 females, as well as when considering deme sizes that exceed 7 males and 7 females. Simulations were run for 2000 generations and infection frequency was recorded for each deme at intervals of 10 generations. We considered an infection to have invaded if it was still present at the end of the simulation run (generation 2000). Simulations were replicated 1000 times for any combination of parameters.

#### Effects of dispersal rate and deme size in an island model

In a first set of simulations, we analyzed the dynamics of infection as a function of host migration rate and deme size in an island model of population structure. In these simulations, the population was sub-divided into demes of sizes *N *= {4, 6, 12, 20, 60, 90, 180}. Since the sex ratio was assumed to be even in our simulations, these deme sizes are equivalent to 2, 3, 6, 10, 30, 45, and 90 reproductive females. With a total population size of 1800, the array of deme sizes imply deme numbers of *n*_*d *_= {450, 300, 150, 90, 30, 20, 10}. Demes were linked by male and female migration at equal rates. The range of migration rates used was *m *= {0.01, 0.05, 0.1, 0.2}. We ran simulations for all combinations of migration rate *m *and deme size *N*.

**Figure 2:**
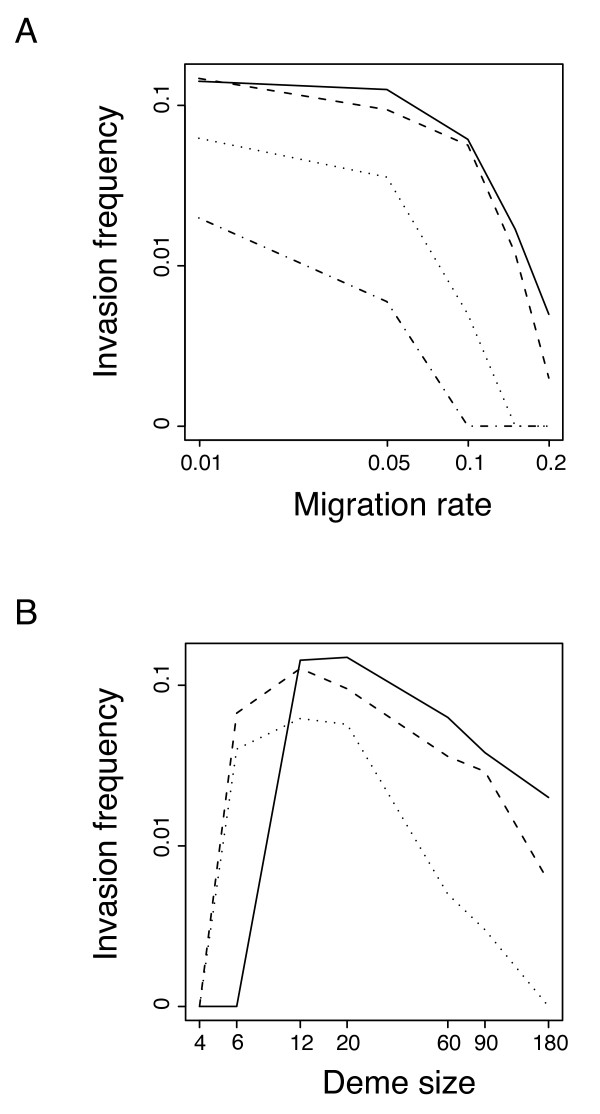
**Simulation results on the effect of population structure on CI-invasion frequency**. The figure shows A, the frequency of invasion as a function of migration rate for deme sizes of N = 12 (solid line), N = 20 (dashed line), N = 60 (dotted line) and N = 180 (dashed-dotted line) and B, the frequency of invasion as a function of deme size for host migration rates of *m *= 0.01 (solid line), *m *= 0.05 (dashed line), and *m *= 0.1 (dotted line).

Frequencies of invasion (i.e., the proportions of replicate simulation runs in which the infection was present after 2000 generations) varied with both deme size and migration in a way that fit the predictions of our analytical model (Fig. 2). While increasing host migration rate lead to a decline in invasion frequency (Fig. 2A), infections were more likely to persist when deme size was intermediate (Fig. 2B). Figure 2 shows that population sub-division (restricted migration) allows CI infection to invade populations it would not have invaded under panmixia. For example, with deme sizes of N = 180, infection can sometimes invade with migration rates of *m *= 0.01 and *m *= 0.05, but not with *m *= 0.1 and above, which for ten demes corresponds to panmixia.

**Figure 3:**
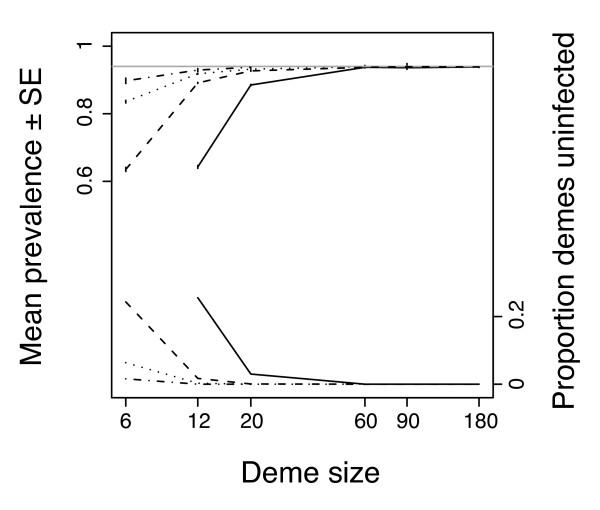
**Simulation results on the mean population prevalence and between-deme polymorphism in infection**. Mean population prevalence (± SE) and the proportion of demes infected as a function of deme size in simulations with equal migration rate in males and females. Results are shown for host migration rates of *m *= 0.01 (solid line), *m *= 0.05 (dashed line), *m *= 0.1 (dotted line), and *m *= 0.2 (dashed-dotted line). The grey line indicates the equilibrium frequency of *p*_*e *_= 0.94 predicted for a panmictic population of infinite size.

In addition to its effect on the probability of spread, population sub-division also influenced the equilibrium frequency they attained. Generally, infections attained a higher equilibrium frequency (prevalence) under conditions that minimized the amount of local genetic drift. Thus, prevalence in populations with strong drift (low migration rate and/or small deme size) remained considerably lower than the value predicted for panmixia (*p*_*e *_= 0.94) at which populations with weak drift (large demes and/or high rates of migration) stabilized (Fig. 3). The low prevalence in high-drift populations is not due to a failure to attain equilibrium. Rather, low prevalence is the result of two mechanisms. First, drift causes prevalence to fluctuate around the equilibrium of *p*_*e *_= 0.94. Because infection frequency cannot exceed 100%, large stochastic fluctuations in populations with strong drift will tend to decrease mean prevalence. Second, under conditions of strong local drift, population-wide mean prevalence is lowered by the total loss of infection in some demes (Fig. 3). For instance, in the two cases showing the lowest mean prevalence (*N *= 12 with *m *= 0.01, and *N *= 6 with *m *= 0.05), around a quarter of demes had lost infection while the prevalence in the remaining infected demes was 86–88% (compared to a population-wide prevalence of about 64%). Additional simulations have shown that such polymorphism is stable over large time-spans (more than 5,000 generations).

#### Effects of dispersal rate and deme size in a stepping stone model

The island model used in the analytical model and the above simulations is convenient from a modelling point of view, but is representative of few, if any, natural situations. Indeed, it assumes that all demes of a population are equidistant, while in natural populations, demes tend to be arranged in a geographic pattern that causes some pairs of demes to be further apart than others. Accordingly, some demes are more likely to exchange migrants than others. In order to assess the influence of geography on the spread of CI, we performed a set of simulations in a simple stepping stone setting, in which the demes were arranged on a circle. Migration occurred between neighbouring demes along the circle. Simulations were run for deme sizes of N = {4, 6, 12, 20, 60, 180} (deme numbers *n*_*d *_= {450, 300, 150, 90, 30, 10}) and migration rates of *m *= {0.01, 0.05, 0.1, 0.2, 0.4, 0.6}.

**Figure 4:**
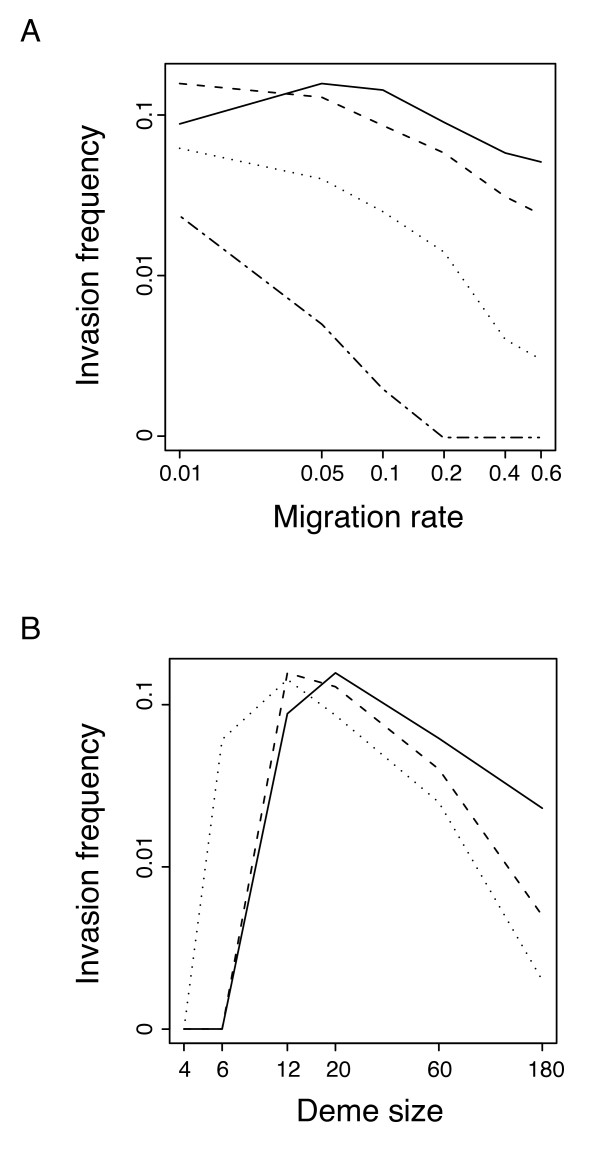
**Simulation results on the effect of population structure on CI-invasion frequency in a stepping stone model**. The figure shows the frequency of invasion, A, as a function of migration rate for deme sizes of N = 12 (solid line), N = 20 (dashed line), N = 60 (dotted line) and N = 180 (dashed-dotted line) and B, as a function of deme size for host migration rates of *m *= 0.01 (solid line), *m *= 0.05 (dashed line), and *m *= 0.1 (dotted line). See Figure 2 for the results with an island model.

Figure 4 shows the frequency of invasion observed in these simulations as a function of migration rate (4A) and deme size (4B). Overall, the results are qualitatively equivalent to those obtained under the island model. Thus, the frequency of CI-invasion tends to decrease with increasing host migration rate. Furthermore, invasion frequency is highest for intermediate deme size. However, comparing Figures 2 and 4 shows that there are quantitative differences between the island model and the stepping stone model. In particular, the explicit geographical arrangement of demes in the stepping stone model appears to increase the probability of CI spread with high rates of host migration. This difference could arise because the limiting migration to adjacent demes in the stepping stone model reduces the degree to which infection is diluted across the entire population, making it more likely that the infection frequency will remain above the infection threshold in groups of neighbouring demes.

#### Infection dynamics as a function of male and female dispersal rates

In a third set of simulations, we addressed the relative importance of male and female dispersal rates for the spread of CI infections. We ran simulations for all combinations of male and females migration rates of *m *= {0.01, 0.05, 0.1, 0.2}. Population sub-division was fixed to *n*_*d *_= 30 and *N *= 60.

**Figure 5:**
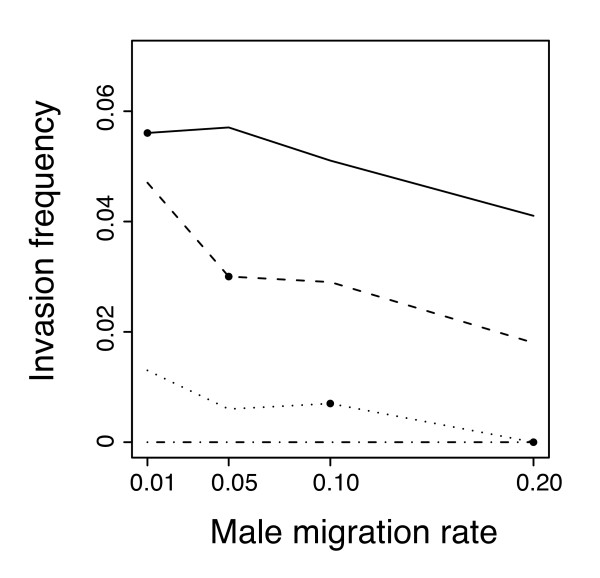
**Simulation results on the effect of sex-specific dispersal rates on CI-invasion frequency**. Invasion frequency is shown for different combinations of male migration rate (on the abscissa) and female migration rate (*mf *= 0.01: solid line; *m*_*f *_= 0.05: dashed line; *m*_*f *_= 0.1: dotted line; *m*_*f *_= 0.2: dashed-dotted line). Dots indicate combinations of equal male and female migration.

The parasites being maternally transmitted, female dispersal was a major determinant of infection dynamics. As shown in Figure 5, the frequency of CI invasion decreased with female dispersal rate. However, male migration rate also had an effect, in that female philopatry favoured the spread of infection more when males dispersed at a low or moderate rate and less if male dispersed at a high rate.

## Discussion

In this paper we have investigated the effect of host population structure on the spread of incompatibility-inducing symbionts. Our analyses demonstrate that population structure can greatly facilitate the spread of CI. In our simulations, infections spread from being present in a single individual of each sex (*p*_*f *_= *p*_*m *_= 1/900) to an equilibrium frequency of *p *≈ 0.94 with probabilities as high as 15% (cf. Figs. 2 and 4), despite the relatively benign level of incompatibility used (*B *= 0.5). These results suggest that the invasion threshold predicted by panmictic models does not represent a major barrier to invasion of CI into structured populations. If regular opportunities exist for horizontal transmission of infections into the populations, the spread of infection is only a matter of time. Population sub-division, which can be assumed to affect most natural populations to some degree, could thus provide the key to understanding the high incidence of CI-inducing symbiont infections that has been observed in arthropods, and in particular insects [18].

Both the model and the simulations consistently showed that the rate of invasion of CI-symbionts varies according to both parameters of host population structure considered in our study, deme size and migration rate. The latter of these parameters affects infection in a straightforward way; high levels of host migration will result in the dilution of infection out of a deme into the population at large and therefore decrease the probability of successful invasion. Furthermore, CI spread relies on limited dispersal of both males and females, in order to assure that infected females enjoy an increased relative fitness as a result of uninfected females suffering incompatible matings. The other parameter, deme size, shows an interesting non-monotonous effect whereby CI-infections invade easiest when deme size is intermediate. Under these conditions, local genetic drift creates heterogeneity in infection among the local breeding population that guarantees a high proportion of incompatible matings and hence a large selective advantage for infection. Small deme size does not favour the spread of infection for two reasons. First, strong drift results in the frequent elimination of infection shortly after the initial inoculation of a population. Second, in the event that infection is not lost but fixed in one deme, it is subject to negative between-deme selection because the fecundity cost of infection reduces the productivity of infected demes. Large deme sizes, in turn, are disadvantageous because local drift is not strong enough to overcome the fitness disadvantage that CI-infections suffer at low frequencies due to the cost of infection.

As outlined above, the non-monotonous dependence of invasion probability on deme size results partly from selection against demes fixed for either the uninfected or the infected state. Infected demes lose out in between-deme competition because their productivity is reduced by the cost of infection. Accordingly, the low invasion success of a CI strategy in populations with small demes is related to the fact that infection reduces fecundity irrespectively of whether CI results in harm to uninfected individuals or not. Invasion success in small demes is expected to be higher for agents (be it genes or symbionts) in which the fecundity cost is conditional on the expression of the harming act (such as CI). This is the situation usually considered in models of the evolution of spiteful behaviour [24,25]. In these models, invasion of spiteful traits has been found to decrease monotonously with deme size.

The simulation results presented here demonstrate not only that population sub-division affects CI-dynamics, but also that this effect varies according to the population model considered. Thus, host migration had a stronger negative effect on CI invasion in the island model than in the stepping-stone model. This quantitative difference can be explained by a lesser degree of dilution of infection in the geographically explicit stepping stone model. Thus, local migration in this population setup resembles diffusion and is expected to result in a spatial correlation of infection frequency, with adjacent demes being more similar in infection than demes further apart. Accordingly, migration out of infected demes will drive up infection frequency in adjacent demes very effectively and is more likely to bring infection frequency in the range of positive selection for infection. The flipside of localized migration is, however, that the spread of infection across the population as a whole proceeds more slowly and more generations are needed to reach the equilibrium prevalence in the population as a whole (data not shown).

Our prediction that population sub-division can favour the spread of a CI infections differs diametrically from that of Wade and Stevens [21]. These authors found that population structure impedes the spread of CI. A closer look at the two models reveals that this discrepancy is rooted in the different ways in which population structure is implemented in the life-cycle underlying the models. Wade and Stevens' model assumes that reproduction takes place within groups. Offspring produced in different matings groups then undergo complete dispersal and compete at the level of the entire population [hard selection sensu [26]], before being partitioned in groups again for the next round of reproduction. Population structure is implemented as variance in infection frequency between mating groups, which is a fraction of the total variance in infection frequency *p*(1-*p*). Importantly, the portion of the total variance in infection that is attributed to the between-deme component is arbitrarily determined by a parameter. Accordingly, between- and within-deme variance are both assumed to vary completely independently of the migration rate and are thus independent of the geographical structure of the population. In this setting, increasing the variance in infection between mating groups will necessarily be disadvantageous for the spread of infection, because it is associated with a more homogeneous infection within demes and hence a reduced frequency of incompatible matings. The spread of CI is further compromised by the assumption that competition occurs at the level of the entire population. Global competition reduces the net advantage of CI because fitness is measured by comparing the fecundity of infected females to the population average rather than the deme average. As a consequence, a high frequency of incompatible matings within some individual demes will only generate a small increase in the relative fitness of infected females across the entire population. In our model, population structure is not imposed as an external parameter but arises naturally as a consequence of limited dispersal between permanent demes. Both reproduction and competition take place within demes, but separated by an episode of dispersal. With such a setting, intermediate between hard and soft selection, population structure affects both the frequency of incompatible matings and the probability that infected individuals will benefit from the increase in relative fitness brought about by CI. Our model shows that, if implemented in this natural way, population sub-division is conducive to the spread of CI-symbionts because the benefit of CI is likely to go to infected individuals rather than being diluted in the population at large. Under the assumption that competition occurs before dispersal (pure soft selection), we expect that population sub-division would be even more in favor of the spread of CI than under our life cycle because competition would occur only locally, thus increasing the benefits of reducing the fecundity of neighbouring uninfected females. This suggests that our results are qualitatively robust to changes of assumptions on the timing of dispersal.

The analytical model presented here assumes that individuals perform a large number of random matings within demes. Violations of this assumption due to systematic inbreeding or inbreeding avoidance alter the dynamics of an incompatibility-inducing mutant. Inbreeding reduces the expected number of incompatible matings and hence impedes the spread of CI. Outbreeding, on the contrary, increases the number of incompatible matings and promotes the spread of infection [27]. These effects of the mating system are expected to occur irrespective of population structure and hence add to the effects of dispersal rate and deme size predicted here. However, population structure is expected to have an influence on the quantitative difference between the frequency change with outbreeding/inbreeding and that with random mating. Both the advantage of outbreeding and the disadvantage of inbreeding are subject to the presence of diversity in infection within the mating populations. Under conditions of strong drift, the mating system can therefore be expected to have a small effect on infection dynamics.

In addition to a simple mating system, random mating, our model and simulations assumed non-overlapping generations. While this assumption might hold for some real-world cases, the majority of species show some degree of overlap between generations. Population age structure has been suggested to negatively affect the dynamics of CI and impeding infection spread [28], although the assumptions of this model are unclear. We have not extended our model in this direction but we would expect that including age structure would have a positive effect on the probability of CI spread. With overlapping generations, only a fraction of the breeding population is replaced at every generation. As a consequence, increasing age structure will, for a given deme size, reduce the part of the breeding population that has just reached adulthood and potentially migrated. Thus, age structure will tend to reduce the effective migration rate and accentuate population sub-division, thereby increasing the probability of CI spread (cf. Fig. 2A).

The predictions generated by our model and simulations have several implications for empirical research on CI-inducing symbionts. First, they generate the testable prediction that symbionts causing CI should be relatively more frequent in species or populations that show a higher degree of sub-division. Furthermore, our results on overall prevalence (Fig. 3) highlight potential problems for obtaining meaningful estimates of prevalence. Most natural hosts populations will be sub-divided and potentially subject to strong genetic drift. Accordingly, the effective population size was found to be around ten times smaller than the numerical population size in a large survey of data on wild animal populations [29]. Under conditions of local drift, measures of prevalence should be based on screens for infection in several demes, because strong drift can generate local variation in infection frequency. A wide sampling screen is the more important the lower the migration rate of hosts and the smaller the size of local breeding populations.

Finally, our results are of fundamental importance with regards to the exploitation of CI as a drive-systems for genetic manipulation of natural populations of arthropods. Introducing transgenes into natural populations can be desirable for a number of reasons, the most important of which is the aim of controlling the transmission of vector-borne disease such as malaria. The realization that CI can bring about the rapid spread of symbiont infection in large host populations has led to the proposition of using the power of this system to drive transgenes through a host population [e.g., [30,31]]. However, previous analyses of the dynamics of such a system based on a panmictic model suggested that large numbers of genetically modified organisms would be required in order to overcome the invasion threshold to achieve successful spread of the construct [31]. Our results suggest that this limitation applies to a much lesser degree to situations in which vector populations are sub-divided. As shown in Figures 2 and 4, infections in highly subdivided populations spread with reasonable probability, even if populations are inoculated with a single pair released in one deme. The probability of successful invasion is expected to be much increased if the number of released individuals is large relative to the size of local demes. By reversing their logic, the predictions of our model further suggest that invasion success could be increased by artificially limiting host migration. A combined strategy of local release of transgenic hosts combined with, for instance, the pesticide treatment of adjacent demes would help the establishment of infection in the focal deme, creating a base for subsequent spread through the remainder of the population.

## Methods

All methods are described in the main text. Additional information on the derivation of the analytical model is provided in the Appendix (Additional file 1).

## Conclusion

Our work has shown that population sub-division greatly facilitates the spread of CI-inducing symbionts and allows infections to invade large populations, even when initially rare. Our study therefore helps to reconcile the high incidence of CI-infections across arthropod species with the considerable barriers to invasion predicted by models of CI-dynamics based on large panmictic populations. Furthermore, our predictions validate the use of CI-systems for the genetic manipulation of natural populations. Indeed, sub-divided populations of hosts could be successfully infected with relatively small numbers of genetically engineered CI-agents, in particular if gene flow can be artificially restricted during the early periods of invasion.

## Authors' contributions

MR conceived the research, MR, LL, and FG performed the research. MR, LL, and FG wrote the paper.

## Supplementary Material

Additional file 1 The Appendix contains a detailed derivation of the analytical model. Click here for file
