# Supplementary material for: The spread of incompatibility-inducing parasites in sub-divided host populations
Source: BMC Evol Biol. 2008 May 6;8:134. doi: 10.1186/1471-2148-8-134 (PMC2396168; doi:10.1186/1471-2148-8-134)
Supplement: Additional file 1 — The Appendix contains a detailed derivation of the analytical model. [file 1471-2148-8-134-S1.pdf]

Appendix

In this appendix, we provide a description of our model predicting the dynamics of CI-infections in structured populations. Infection is modelled as a two-state variable, with each individual being in one of the two states: "infected" or "uninfected". We designate by p the frequency of infected individuals among hosts in the population (hereafter referred to simply as the "infection frequency"). Because selection depends on mating frequencies and thus on the interaction between the sexes, we distinguish infection frequency in females, designated by p_{f}, from that in males, designated by p_{m}. We assume that infection is purely maternally transmitted and that organisms are diploid; since the sex-ratio is even we have $p_{\mathrm{f}} = p_{\mathrm{m}}$. Therefore, the change in frequency of infection is sufficiently described by the average change in frequency of infection in the population among female hosts, p_{f}, which can be written as

$$\Delta p_{\mathrm{f}} = \mathrm{E}\left[w_{ij} p_{\mathrm{f}(ij)}\right] - p_{\mathrm{f}}, \tag{1}$$

where w_{ij} is the expected number of successful offspring of female i reproducing in deme j, $p_{\mathrm{f}(ij)}$ is the frequency of infection in this female (0 or 1), and $\mathrm{E}[\cdot]$ denotes the average over all demes in the population and all females within a deme. Since the population is assumed to be of constant size, the mean fitness is equal to one ($\mathrm{E}[w_{ij}] = 1$). To keep our analysis simple, we assume that w_{ij} has a common form for all demes (i.e., the environment is homogeneous) and that the number of demes in the population is very large.

The fitness w_{ij} of female i in deme j depends on its expected number of offspring produced (i.e., fecundity), itself a function of infection frequency in female i and in males of deme j. In order to obtain an explicit expression for fecundity, we extend the expression of parasite fecundity for panmictic population given by Turelli (1994), and, without loss of generality, we consider only the fecundity of individuals relative to the fecundity of uninfected individuals. The relative fecundity of female i in deme j will be written as $1 + f_{ij}$, where f_{ij} represents the decrement of fecundity due to infection, composed of the effect of the genotype of the female herself and the genotype of the males with which she has mated. According to our life cycle assumptions (see main text), the relative fecundity is given by

$$1 + f_{ij} = 1 - Cp_{\mathrm{f}(ij)} - B(1 - p_{\mathrm{f}(ij)})p_{\mathrm{m}(j)}, \tag{2}$$

where $p_{\mathrm{m}(j)} = \frac{1}{N}\sum_i p_{\mathrm{m}(ij)}$ is the average frequency of infected males in deme j, and $p_{\mathrm{m}(kj)}$ is the frequency of infection (0 or 1) in male k from deme j. Averaging eq. 2 over all females

within deme j gives

$$\begin{aligned} 1+f_j &= \frac{1}{N}\sum_i (1+f_{ij}) \\ &= 1 - Cp_{\mathrm{f}(j)} - B(1-p_{\mathrm{f}(j)})p_{\mathrm{m}(j)}, \end{aligned} \tag{3}$$

where $p_{\mathrm{f}(j)} = \frac{1}{N}\sum_i p_{\mathrm{f}(ij)}$ is the average frequency of infected females in deme j. Finally, the average of eq. 3 over all demes in the population is

$$\begin{aligned} 1+f &= \frac{1}{n_{\mathrm{d}}}\sum_j (1+f_j) \\ &= 1 - Cp_{\mathrm{f}} - Bp_{\mathrm{m}} + B\mathrm{E}\left[p_{\mathrm{m}(j)}p_{\mathrm{f}(j)}\right], \end{aligned} \tag{4}$$

where the number n_{d} of demes in the population is assumed to be very large ($n_{\mathrm{d}} \to \infty$), and $\mathrm{E}\left[p_{\mathrm{m}(j)}p_{\mathrm{f}(j)}\right]$ is the probability of sampling an infected male and an infected female from the same deme. Due to our assumption of an infinite number of demes, an average fecundity over all demes, excluding a single focal deme, converges to the average fecundity $1+f$ over all demes in the population. For notational simplicity, we will thus use in the following calculations $1+f$ for any average over all demes while excluding a single focal deme.

We now have all the elements to evaluate the fitness function w_{ij}. A number $(1-m)(1+f_{ij})$ of the offspring of female i from group j remain in group j and compete against an average number $(1-m)(1+f_j)$ of offspring produced in that group and an average number $m(1+f)$ of immigrant offspring produced in different groups. A number $m(1+f_{ij})$ of the offspring of female i from group j disperse and enter in competition against an average number $(1+f)$ of offspring. Collecting all terms, the fitness of female i from group j becomes

$$w_{ij} = \frac{(1-m)(1+f_{ij})}{(1-m)\,(1+f_i) + m(1+f)} + \frac{m(1+f_{ij})}{1+f}, \tag{5}$$

which is of the same form as the fitness function of previous and closely related models (Roze and Rousset, 2003, 2004; Lehmann et al., 2007)

We now invoke weak selection, an assumptions that is often endorsed in social evolutionary theory or population genetics (e.g., Hamilton, 1964; Nagylaki, 1993; Taylor, 1996; Roze and Rousset, 2003, 2004; Kirkpatrick et al., 2002; Grafen, 2006), and implies that the phenotypic effects of infection, C and B, are of small order δ, where δ can be thought off as

the largest of the two effects of infection on fitness. Therefore, f_{ij}, f_j and f will also be of order δ and recalling that for x small we have $y/(1+x) \simeq y(1-x)$, eq. 5 becomes

$$w_{ij} = 1 + f_{ij} - (1-m)^2 f_j - (1-(1-m)^2)f + O(\delta^2), \tag{6}$$

where $O(\delta^2)$ is a remainder of order δ^2. Substituting the fitness function into eq. 1 yields for weak selection

$$\begin{aligned} \Delta p_{\mathrm{f}} &= \mathrm{E}\left[\{f_{ij} - (1-m)^2 f_j - (1-(1-m)^2)f\} p_{\mathrm{f}(ij)}\right] + O(\delta^2) \\ &= \mathrm{E}\left[f_{ij} p_{\mathrm{f}(ij)}\right] - (1-m)^2 \mathrm{E}\left[f_j p_{\mathrm{f}(ij)}\right] - (1-(1-m)^2)\mathrm{E}\left[f p_{\mathrm{f}(ij)}\right]. \end{aligned} \tag{7}$$

Inserting eqs. 2-4 into this expression and noting that $p_{\mathrm{f}(ij)}^2 = p_{\mathrm{f}(ij)}$, the first expectation appearing in eq. 7 becomes

$$\mathrm{E}\left[f_{ij} p_{\mathrm{f}(ij)}\right] = -C p_{\mathrm{f}} \tag{8}$$

while the second expectation in eq. 7 is given by

$$\mathrm{E}\left[f_j p_{\mathrm{f}(ij)}\right] = -C\mathrm{E}\left[p_{\mathrm{f}(j)} p_{\mathrm{f}(j)}\right] - B\{\mathrm{E}\left[p_{\mathrm{m}(j)} p_{\mathrm{f}(j)}\right] - \mathrm{E}\left[p_{\mathrm{m}(j)} p_{\mathrm{f}(j)} p_{\mathrm{f}(j)}\right]\}, \tag{9}$$

and finally

$$\mathrm{E}\left[f p_{\mathrm{f}(ij)}\right] = p_{\mathrm{f}}\left(-C p_{\mathrm{f}} - B\{p_{\mathrm{m}} - \mathrm{E}\left[p_{\mathrm{m}(j)} p_{\mathrm{f}(j)}\right]\}\right). \tag{10}$$

Substituting the last three terms into eq. 7, the change in infection frequency becomes

$$\begin{aligned} \Delta p_{\mathrm{f}} = -C\, p_{\mathrm{f}} + (1-m)^2 \left(C\, \mathrm{E}\left[p_{\mathrm{f}(j)} p_{\mathrm{f}(j)}\right] + B\{\mathrm{E}\left[p_{\mathrm{m}(j)} p_{\mathrm{f}(j)}\right] - \mathrm{E}\left[p_{\mathrm{m}(j)} p_{\mathrm{f}(j)} p_{\mathrm{f}(j)}\right]\}\right) \\ + p_{\mathrm{f}}\left(1-(1-m)^2\right)\left(C p_{\mathrm{f}} + B\{p_{\mathrm{m}} - \mathrm{E}\left[p_{\mathrm{m}(j)} p_{\mathrm{f}(j)}\right]\}\right), \end{aligned} \tag{11}$$

where the expectations of pairs and triplets of infection frequencies are probabilities that pairs and triplets of individuals carry the infection. These probabilities will be evaluated by following the rationale usually employed for evaluating probabilities of identity between homologous genes in the infinite island model of dispersal, where two infection strains sampled from different demes are considered as genealogically independent, and thus bear no identity by descent (e.g., Perrin and Mazalov, 2000; Whitlock, 2002; Cherry and Wakeley, 2003; Roze and Rousset, 2003). For simplicity, we will also use the notation $p_{\mathrm{f}} = p_{\mathrm{m}} = p$.

We start by evaluating identities between pairs of infection statuses. The probability $\mathrm{E}\left[p_{\mathrm{f}(j)} p_{\mathrm{f}(j)}\right]$ that two females randomly sampled with replacement from the same deme are

both infected comprises two events. First, the two females can have inherited their infection from an infected common ancestor. In the infinite island model this implies that, when looking backwards in time, the ancestral lineages of the two females' infection strains have always stayed in the same deme and coalesced in a local ancestor. If we call the probability of this happening $F_{\mathrm{ST}}^{\mathrm{R}}$, the probability of sharing an infection by descent is $F_{\mathrm{ST}}^{\mathrm{R}}p$, where the weight of the infection's frequency in the population, p, expresses the probability that the common ancestor carried the infection. Alternatively, the two females can have inherited their infections independently. This event occurs with the probability of non-coalescence between the two females' infection strains, $1 - F_{\mathrm{ST}}^{\mathrm{R}}$, weighted by the probability of two independent individuals sampled at random from the population being infected, p^2. Hence, the total probability that both females share the same infection statuses is

$$\mathrm{E}\left[p_{\mathrm{f}(j)}p_{\mathrm{f}(j)}\right] = F_{\mathrm{ST}}^{\mathrm{R}}p + (1 - F_{\mathrm{ST}}^{\mathrm{R}})p^2, \tag{12}$$

where

$$F_{\mathrm{ST}}^{\mathrm{R}} = \frac{1}{N} + \left(\frac{N-1}{N}\right) F_{\mathrm{ST}}. \tag{13}$$

In this expression, $1/N$ is the probability of sampling twice the same female and F_{ST} is the probability of coalescence of two distinct infection lineage that is given by

$$F_{\mathrm{ST}} = \sum_{t=1}^{\infty}(1-m)^{2t}\left(1 - \frac{1}{N}\right)^{t-1}\frac{1}{N}, \tag{14}$$

where $(1-m)^{2t}$ is the probability that the two infection lineages remained in the same deme for at least t generations and its factor is the probability that the lineages coalesced precisely at that time. In practice, it is more convenient to evaluate F_{ST} from recurrence equations (e.g., Hartl and Clark, 1997; Perrin and Mazalov, 2000; Gillespie, 2004; Roze and Rousset, 2003), which is given at equilibrium by

$$F_{\mathrm{ST}} = (1-m)^2\left(\frac{1}{N} + \frac{(N-1)}{N}F_{\mathrm{ST}}\right), \tag{15}$$

Following a reasoning along the same lines as those detailed above, we can calculate the probability $\mathrm{E}\left[p_{\mathrm{m}(j)}p_{\mathrm{f}(j)}\right]$ that a randomly sampled pair of one male and one female both bear the infection. This probability is given by

$$\mathrm{E}\left[p_{\mathrm{m}(j)}p_{\mathrm{f}(j)}\right] = F_{\mathrm{ST}}p + (1 - F_{\mathrm{ST}})p^2. \tag{16}$$

We now turn to the evaluation of the identity between triplets infection statuses. The probability $\mathrm{E}\left[p_{\mathrm{m}(j)}p_{\mathrm{f}(j)}p_{\mathrm{f}(j)}\right]$ that one male and two females sampled with replacement from

the same deme all bear the infection comprises three events. First, with probability $F_3^{\mathrm{R}}p$ the three individuals share their infection statuses by common descent because all three gene lineages coalesce in a local common ancestor (with a probability called F_3^{R}) who bore the infection strain (with probability p). Second, the three individuals can all be infected because two of them share their infection by common local descent while the third individual is part of an immigrant lineage of the infected type. This occurs with probability $(F_{\mathrm{ST}}^{\mathrm{R}}+2F_{\mathrm{ST}}-3F_3^{\mathrm{R}})p^2$ where $F_{\mathrm{ST}}^{\mathrm{R}}$ is the probability of coalescence of genes in two females sampled with replacement in a deme and F_{ST} is the probability of coalescence of two gene, one in a male and one in a female. The probability F_3^{R} has to be subtracted in order to exclude cases in which not only one pair of strain lineages but all three coalesce. Third, with a probability complementary to the two just described, the three strains lineages do not coalesce, in which case they all bear the infection status with probability p^3. Thus,

$$\begin{aligned}\mathrm{E}\left[p_{\mathrm{m}(j)}p_{\mathrm{f}(j)}p_{\mathrm{f}(j)}\right] = F_3^{\mathrm{R}}p + \left(F_{\mathrm{ST}}^{\mathrm{R}} + 2F_{\mathrm{ST}} - 3F_3^{\mathrm{R}}\right)p^2 \\ + \left(1 - F_{\mathrm{ST}}^{\mathrm{R}} - 2F_{\mathrm{ST}} + 2F_3^{\mathrm{R}}\right)p^3,\end{aligned} \tag{17}$$

where

$$F_3^{\mathrm{R}} = \frac{1}{N}F_{\mathrm{ST}} + \left(\frac{N-1}{N}\right)F_3, \tag{18}$$

is the probability of identity by descent between three strains, two of which are sampled with replacement and

$$F_3 = (1-m)^3\left(\frac{1}{N^2} + \frac{3(N-1)}{N^2}F_{\mathrm{ST}} + \frac{(N-2)(N-1)}{N^2}F_3\right). \tag{19}$$

is the equilibrium probability of identity between three strains sampled without replacement from the same deme after dispersal.

Inserting all the probabilities of identity in state (eq. 12, eq. 16 and eq. 17) into the equation of infection frequency change (eq. 11), we obtain after simplification that

$$\Delta p = p(1-p)\left[-C(1-F_{\mathrm{ST}}) + B(1-m)^2\left(F_{\mathrm{ST}} - F_3^{\mathrm{R}}\right) + pB\{1-g(N,m)\}\right]. \tag{20}$$

where

$$g(N,m) = 2F_{\mathrm{ST}} + (1-m)^2(F_{\mathrm{ST}} - 2F_3^{\mathrm{R}}). \tag{21}$$

From these equations we can express the condition for infection to spread when rare ($p \to 0$) in terms of the cost-to-benefit ratio as

$$\frac{C}{B} < (1-m)^2\left(\frac{F_{\mathrm{ST}} - F_3^{\mathrm{R}}}{1 - F_{\mathrm{ST}}}\right). \tag{22}$$

References

Cherry, J. L. and J. Wakeley. 2003. A diffusion approximation for selection and drift in a subdivided population. *Genetics* 163:421–428.

Gillespie, J. H. 2004. Population genetics: a concise guide. Johns Hopkins, Baltimore & London.

Grafen, A. 2006. Optimization of inclusive fitness. *Journal of Theoretical Biology* 238:541–563.

Hamilton, W. D. 1964. The genetical evolution of social behaviour, I. *Journal of Theoretical Biology* 7:1–16.

Hartl, D. and A. G. Clark. 1997. Principles of population genetics. Sinauer, Massachusetts.

Kirkpatrick, M., T. Johnson, and N. Barton. 2002. General models of multilocus evolution. *Genetics* 161:1727–1750.

Lehmann, L., F. Rousset, D. Roze, and L. Keller. 2007. Strong reciprocity or strong ferocity? A population genetic view of the evolution of altruistic punishment. *The American Naturalist* 170:21–36.

Nagylaki, T. 1993. The evolution of multilocus systems under weak selection. *Genetics* 134:627–647.

Perrin, N. and V. Mazalov. 2000. Local competition, inbreeding and the evolution of sex-biased dispersal. *The American Naturalist* 155:116–127.

Roze, D. and F. Rousset. 2003. Selection and drift in subdivided populations: A straightforward method for deriving diffusion approximations and applications involving dominance, selfing and local extinctions. *Genetics* 165:2153–2166.

Roze, D. and F. Rousset. 2004. The robustness of Hamilton's rule with inbreeding and dominance: Kin selection and fixation probabilities under partial sib mating. *American Naturalist* 164:214–231.

Taylor, P. D. 1996. Inclusive fitness arguments in genetic models of behaviour. *Journal of Mathematical Biology* 34:654–674.

Turelli, M. 1994. Evolution of incompatibility-inducing microbes and their hosts. *Evolution* 48:1500–1513.

Whitlock, M. C. 2002. Selection, load and inbreeding depression in a large metapopulation. *Genetics* 160:1191–1202.
